# Distinct Modulation of *I*_h_ by Synaptic Potentiation in Excitatory and Inhibitory Neurons

**DOI:** 10.1523/ENEURO.0185-24.2024

**Published:** 2024-11-12

**Authors:** Lotte J. Herstel, Corette J. Wierenga

**Affiliations:** ^1^Biology Department, Faculty of Science, Utrecht University, Utrecht 3584 CH, the Netherlands; ^2^Donders Institute for Brain, Cognition and Behaviour, Radboud University, Nijmegen 6525 AJ, the Netherlands

**Keywords:** cell-type–specific regulation, dendritic integration, feedforward inhibition, HCN channels

## Abstract

Selective modifications in the expression or function of dendritic ion channels regulate the propagation of synaptic inputs and determine the intrinsic excitability of a neuron. Hyperpolarization-activated cyclic nucleotide–gated (HCN) channels open upon membrane hyperpolarization and conduct a depolarizing inward current (*I*_h_). HCN channels are enriched in the dendrites of hippocampal pyramidal neurons where they regulate the integration of synaptic inputs. Synaptic plasticity can bidirectionally modify dendritic HCN channels in excitatory neurons depending on the strength of synaptic potentiation. In inhibitory neurons, however, the dendritic expression and modulation of HCN channels are largely unknown. In this study, we systematically compared the modulation of *I*_h_ by synaptic potentiation in hippocampal CA1 pyramidal neurons and stratum radiatum (sRad) interneurons in mouse organotypic cultures. *I*_h_ properties were similar in inhibitory and excitatory neurons and contributed to resting membrane potential and action potential firing. We found that in sRad interneurons, HCN channels were downregulated after synaptic plasticity, irrespective of the strength of synaptic potentiation. This suggests differential regulation of *I*_h_ in excitatory and inhibitory neurons, possibly signifying their distinct role in network activity.

## Significance Statement

Learning changes how information is processed in neuronal circuits. This occurs via alterations in synaptic connections and intrinsic excitability of neurons. Here we examined how synaptic changes affect properties of HCN channels, which are important ion channels for intrinsic excitability. We found that strong synaptic potentiation leads to opposite changes in HCN channels in CA1 pyramidal neurons and stratum radiatum (sRad) interneurons. We speculate that this reflects their differential role in the CA1 network. An upregulation of HCN channels in pyramidal neurons results in a decreased excitability, which limits overall network excitation. In contrast, sRad interneurons show downregulation of *I*_h_ and therefore an increased excitability after strong synaptic activation, which will strengthen feedforward inhibition and sharpen activity patterns.

## Introduction

The intrinsic excitability and firing properties of a neuron can be adjusted via selective modifications in the expression or function of specific ion channels (Daoudal and Debanne, 2003; Zhang and Linden, 2003; Debanne et al., 2019). Firing properties are mostly determined by voltage-dependent ion channels in the soma, while ion channels within dendrites regulate the spatial and temporal integration of synaptic inputs along the dendrites (Hoffman et al., 1997; Migliore and Shepherd, 2002; Nolan et al., 2004; Magee and Johnston, 2005; Branco and Häusser, 2010; Oz et al., 2022). Plasticity of neuronal excitability critically contributes to learning and adaptation (Zhang and Linden, 2003; Shah et al., 2010; Hengen et al., 2016; Debanne et al., 2019), memory (Losonczy et al., 2008; Makara et al., 2009), social learning (Gao et al., 2017), and fear conditioning (Carzoli et al., 2023).

**Table 1.** Statistics

Figures	Data structure	Type of test	Sample size (n)	*p* value	Power
1*D*	Normal distribution	Paired Student’s *t* test	11 cells, 11 slices, 8 mice	*p* < 0.0001	Mean diff., −8.7 ± 1.0; 95% CI, −11.0 to −6.4
1*E*	Normal distribution	Paired Student’s *t* test	11 cells, 11 slices, 8 mice	*p* = 0.005	Mean diff., −20.3 ± 5.6; 95% CI, −32.7 to −7.9
1*F* *V*_sag_	Normal distribution	Mixed effects model current *×* condition with Sidaks multiple-comparison test	11 cells, 11 slices, 8 mice	*F*_(6,68)_ = 10.45	−200 pA: mean diff., 6.1 ± 1.1; 95% CI, 3.1 to 9.1
				*p* < 0.0001	−300 pA: mean diff., 7.2 ± 1.1; 95% CI, 4.2–10.2
					−400 pA: mean diff., 8.3 ± 1.1; 95% CI, 5.232–11.28
					−500 pA: mean diff., 9.3 ± 1.1; 95% CI, 6.2–12.4
					−600 pA: mean diff., 9.3 ± 1.1; 95% CI, 6.2–12.4
1*F* *V*_ss_	Normal distribution	Two-way repeated–measure ANOVA current *×* condition	11 cells, 11 slices, 8 mice	*F*_(6,70)_ = 1.35	
				*p* = 0.25	
1*H*	Normal distribution	Two-way repeated–measure ANOVA current *×* condition with Sidaks multiple-comparison test	11 cells, 11 slices, 8 mice	*F*_(6,66)_ = 2.26	−50 pA: mean diff., −3.5 ± 1.1; 95% CI, −6.6 to −0.3; *p* = 0.02
					−100 pA: mean diff., −4.8 ± 1.1; 95% CI, −8.0 to −1.6; *p* = 0.0006
				*p* = 0.048	−150 pA: mean diff., −3.4 ± 1.1; 95% CI, −6.5 to −0.2; *p* = 0.03
1*I*	Normal distribution	Paired Student’s *t* test	11 cells, 11 slices, 8 mice	*p* = 0.008	Mean diff., −122.8 ± 36.8, 95% CI: −204.9 to −40.7
1*J*	Normal distribution	Paired Student’s *t* test	11 cells, 11 slices, 8 mice	*p* < 0.0001	Mean diff., −6.4 ± 1.0; 95% CI, −8.5 to −4.3
1*L*	Normal distribution	Paired Student’s *t* test	11 cells, 11 slices, 9 mice	*p* < 0.0001	Mean diff., −10.8 ± 1.4; 95% CI, −13.9 to −7.6
1*M*	Normal distribution	Paired Student’s *t* test	11 cells, 11 slices, 9 mice	*p* = 0.0001	Mean diff., −28.8 ± 4.8; 95% CI, −39.6 to −18.0
1*N* *V*_sag_	Normal distribution	Two-way repeated–measure ANOVA current *×* condition with Sidaks multiple-comparison test	11 cells, 11 slices, 9 mice	*F*_(6,70)_ = 12.19	−100 pA: mean diff., 4.1 ± 1.1; 95% CI, 0.9–7.3; *p* = 0.004
				*p* < 0.0001	−150 pA: mean diff., 5.7 ± 1.1; 95% CI, 2.5–8.9; *p* < 0.0001
					−200 pA: mean diff., 7.8 ± 1.1; 95% CI, 4.6–11.0; *p* < 0.0001
					−250 pA: mean diff., 9.1 ± 1.1; 95% CI, 5.9–12.2; *p* < 0.0001
					−300 pA: mean diff., 10.8 ± 1.1; 95% CI, 7.6–13.9; *p* < 0.0001
1*N* *V*_ss_		Two-way repeated-measure ANOVA current *×* condition with Sidaks multiple-comparison test	11 cells, 11 slices, 9 mice	*F*_(6,70)_ = 9.39	−100 pA: mean diff., 9.9 ± 3.3; 95% CI, 0.8–18.9; *p* = 0.03
				*p* < 0.0001	−150 pA: mean diff., 13.8 ± 3.3; 95% CI, 4.7–22.9; *p* < 0.0005
					−200 pA: mean diff., 18.0 ± 3.3; 95% CI, 8.9–27.1, *p* < 0.0001
					−250 pA: mean diff., 22.6 ± 3.3; 95% CI, 13.5–31.7; *p* < 0.0001
					−300 pA: mean diff., 28.8 ± 3.3; 95% CI, 19.7–37.9; *p* < 0.0001
1*P*	Normal distribution	Two-way repeated–measure ANOVA current *×* condition with Sidaks multiple-comparison test	15 cells, 15 slices, 11 mice	*F*_(6,98)_ = 3.17	−100 pA: mean diff., −3.1 ± 0.7; 95% CI, −4.9 to −1.3; *p* < 0.0001
				*p* = 0.007	
1*Q*	Normal distribution	Paired Student’s *t* test	15 cells, 15 slices, 11 mice	*p* = 0.0005	Mean diff., −61.9 ± 13.7; 95% CI, −91.2 to −32.6
1*R*	Normal distribution	Wilcoxon signed-rank test	11 cells, 11 slices, 8 mice	*p* = 0.002	
2*D*	Non-normal distribution	Wilcoxon signed-rank test	10 cells, 10 slices, 8 mice	*p* = 0.01	
2*F*	Normal distribution	Paired Student’s *t* test	8 cells, 8 slices, 6 mice	*p* = 0.0004	Mean diff., 5.3 ± 0.8; 95% CI, 3.3–7.2
2*I*		Kolmogorov-Smirnov test	1,015 mIPSCs, 7 cells	*p* < 0.0001	
2*J*		Kolmogorov-Smirnov test	1,015 mIPSCs, 7 cells	*p* = 0.008	
2*K*		Kolmogorov-Smirnov test	675 mIPSCs, 9 cells	*p* < 0.0001	
2*L*		Kolmogorov-Smirnov test	675 mIPSCs, 9 cells	*p* = 0.007	
2*M*	Normal distribution	Paired Student’s *t* test	7 cells, 7 slices, 6 mice	*p* = 0.004	Mean diff., 2.0 ± 0.4; 95% CI, 1.0–3.1
2*N*	Normal distribution	Paired Student’s *t* test	7 cells, 7 slices, 6 mice	*p* = 0.36	Mean diff., 0.5 ± 0.5; 95% CI, −0.7 to 1.6
2*O*	Normal distribution	Paired Student’s *t* test	7 cells, 7 slices, 6 mice	*p* = 0.002	Mean diff., 4.0 ± 0.8; 95% CI, 2.2–5.9
2*P*	Non-normal distribution	Wilcoxon signed-rank test	9 cells, 9 slices, 6 mice	*p* = 0.004	
2*Q*	Normal distribution	Paired Student’s *t* test	9 cells, 9 slices, 6 mice	*p* = 0.35	Mean diff., 0.7 ± 0.7; 95% CI, −0.9 to 2.3
2*R*	Normal distribution	Paired Student’s *t* test	9 cells, 9 slices, 6 mice	*p* = 0.01	Mean diff., 5.3 ± 1.7; 95% CI, 1.5–9.2
*a* *	Non-normal distribution	Mann–Whitney *U* test		*p* = 0.054	
3D	Non-normal distribution	Mann–Whitney *U* test	Up: 7 cells, 7 slices, 6 mice; Down: 8 cells, 8 slices, 8 mice	*p* = 0.0003	
3*E* *V*_sag_		Two-way repeated–measure ANOVA current *×* condition with Sidaks multiple-comparison test	6 cells, 6 slices, 5 mice	*F*_(6,33)_ = 2.52 *p* = 0.04	−100 pA: mean diff., 1.7 ± 0.5; 95% CI, 0.2–3.3; *p* = 0.02
					−150 pA: mean diff., 1.9 ± 0.5; 95% CI, 0.4–3.4; *p* = 0.007
3*F* *V*_ss_		Two-way repeated-measure ANOVA current *×* condition with Sidaks multiple-comparison test	6 cells, 6 slices, 5 mice	*F*_(6,33)_ = 11.89 *p* < 0.0001	−100 pA: mean diff., −2.2 ± 0.7; 95% CI, −4.1 to −0.3; *p* = 0.02
					−150 pA: mean diff., −3.6 ± 0.7; 95% CI, −5.5 to −1.7; *p* < 0.0001
					−200 pA: mean diff., −4.5 ± 0.7; 95% CI, −6.4 to −2.6; *p* < 0.0001
					−250 pA: mean diff, −6.0 ± 0.7; 95% CI, −8.0 to −3.9; *p* < 0.0001
					−300 pA: mean diff, −6.4 ± 0.7; 95% CI, −8.5 to −4.4; *p* < 0.0001
3*F* *V*_sag_		Two-way repeated–measure ANOVA current *×* condition with Sidaks multiple-comparison test	7 cells, 7 slices, 7 mice	*p* = 0.81	
3*F* *V*_ss_		Two-way repeated–measure ANOVA current *×* condition with Sidaks multiple-comparison test	7 cells, 7 slices, 7 mice	*p* = 0.18	
3*H*	Normal distribution	Paired Student’s *t* test	7 cells, 7 slices, 6 mice	*p* = 0.056	Mean diff., −1.9 ± 0.8; 95% CI, −3.8 to 0.1
3*I*	Normal distribution	Paired Student’s *t* test	7 cells, 7 slices, 6 mice	*p* = 0.02	Mean diff., 54.0 ± 18.0; 95% CI, 9.9–98.1
3*J*	Normal distribution	Paired Student’s *t* test	8 cells, 8 slices, 8 mice	*p* = 0.011	Mean diff., 1.4 ± 0.4; 95% CI, 0.5–2.4
3*K*	Normal distribution	Paired Student’s *t* test	8 cells, 8 slices, 8 mice	*p* = 0.051	Mean diff., −58.1 ± 23.9; 95% CI, −116.6 to 0.3
4*F*	Normal distribution	One-sample Student’s *t* tests	MP: 5 cells, 5 slices, 4 mice; SP: 6 cells, 6 cells, 6 mice	MP 30′: *p* = 0.21	MP 30′, discr.: 0.6 ± 0.4; 95% CI, −0.6 to 1.8
				SP 30′: *p* = 0.40	SP 30′: discr., −1.0 ± 1.0; 95% CI, −3.7 to 1.7
				MP 60′: *p* = 0.14	MP 60′: discr., 0.9 ± 0.5; 95% CI, −0.5 to 2.3
				SP 60′: *p* = 0.36	SP 60′: discr., −1.7 ± 1.7; 95% CI, −6.0 to 2.6
4*G*	Normal distribution	One-sample Student’s *t* tests	MP: 5 cells, 5 slices, 4 mice	MP 30′: *p* = 0.34	MP 30′: discr., 2.7 ± 2.5; 95% CI, −4.3 to 9.8
				SP 30′: *p* = 0.08	SP 30′: discr., 2.8 ± 1.3; 95% CI, −0.4 to 6.0
			SP: 6 cells, 6 cells, 6 mice	MP 60′: *p* = 0.55	MP 60′: discr., 2.119 ± 3.2; 95% CI, −6.9 to 11.1
				SP 60′: *p* = 0.009	SP 60′: discr., 6.858 ± 1.7; 95% CI, 2.6–11.1
4*H* *V*_sag_	Normal distribution	Two-way repeated–measure ANOVA current *×* condition	11 cells, 11 slices, 7 mice	*F*_(12,140)_ = 0.94	
				*p* = 0.51	
*V*_ss_	Normal distribution	Two-way repeated–measure ANOVA current *×* condition	11 cells, 11 slices, 7 mice	*F*_(12,140)_ = 1.77	
				*p* = 0.06	
4*J*	Normal distribution	Two-way repeated–measure ANOVA	11 cells, 11 slices, 7 mice	*p* = 0.99	
4*K*	Normal distribution	Mixed-effect model current *×* condition	11 cells, 11 slices, 7 mice	*F*_(20,172)_ = 5.33	50 pA 30′ vs 60′: mean diff., 81.9 ± 21.8; 95% CI, 3.9–159.2; *p* = 0.04
				*p* < 0.0001	75 pA pre- vs 60′: mean diff., 116.3 ± 35.9; 95% CI, 6.1–226.5; *p* = 0.04
					125 pA pre- vs 30′: mean diff., 24.7 ± 7.5; 95% CI, 1.6–47.7; *p* = 0.04
					150 pA pre- vs 30′: mean diff., 11.5 ± 3.6; 95% CI, 1.1–21.9; *p* = 0.03
4*L*	Non-normal distribution	Friedman test	11 cells, 11 slices, 7 mice	*p* = 0.11	
4*N*	Normal distribution	One-way ANOVA	7 cells, 7 slices, 5 mice	*p* = 0.27	
4*P*	Normal distribution	One-way ANOVA	7 cells, 7 slices, 5 mice	*p* = 0.12	
*b* *	Non-normal distribution	Wilcoxon signed-rank test	11 cells, 11 slices, 7 mice	*p* = 0.03	

* Correspond to *a* and *b* in the text, indicating statistical tests that are only mentioned in the text.

Contrary to most other voltage-gated ion channels, hyperpolarization-activated cyclic nucleotide–gated (HCN) channels open when the membrane potential is hyperpolarized. HCN channels are permeable to potassium and sodium ions, which means that they conduct a depolarizing inward current (*I*_h_). A substantial fraction of HCN channels is open at rest, resulting in a small depolarization of the resting membrane potential (*V*_rest_). The depolarizing *I*_h_ increases upon hyperpolarization and decreases when the membrane gets depolarized. Therefore *I*_h_ acts to dampen synaptic inputs from both excitatory and inhibitory synapses (Magee, 1999). Because of these distinctive properties, HCN channels are an important contributor to network oscillations (Hu et al., 2009; Zemankovics et al., 2010; Gastrein et al., 2011; Vaidya and Johnston, 2013; Binini et al., 2021). *I*_h_ also influences the threshold for potentiation of synapses (Losonczy et al., 2008; Shah et al., 2010; Carzoli et al., 2023).

HCN channels are abundantly present in the dendrites of excitatory pyramidal neurons, following a gradient with higher density in the distal dendrites (Lörincz et al., 2002; Harnett et al., 2015). The presence of HCN channels in dendrites reduces dendritic excitability (Magee, 1998; Poolos et al., 2002; Campanac et al., 2008), and their specific distribution along the dendrites results in compartment-specific effects of *I*_h_ (Harnett et al., 2015; Mäki-Marttunen and Mäki-Marttunen, 2022). Computational modeling showed that the degree of temporal summation of dendritic inputs is primarily determined by the total number of HCN channels and that local dendritic processing is regulated by their dendritic spatial distribution (Angelo et al., 2007). In the hippocampus, HCN channels can also be found in most GABAergic interneurons (Maccaferri and McBain, 1996; Aponte et al., 2006; Anderson et al., 2011; Sekulić et al., 2015). Differences in subcellular localization and/or properties of HCN channels between inhibitory cell types are shown to affect cell-type–specific firing properties (Lupica et al., 2001; Aponte et al., 2006; Elgueta et al., 2015; Sekulić et al., 2020), synaptic integration (Sammari et al., 2022), and differential involvement in network activity (Zemankovics et al., 2010; Anderson et al., 2011).

Remarkably, HCN channel properties are strongly regulated by multiple intracellular pathways (Poolos et al., 2002, 2006; Concepcion et al., 2021). HCN channel trafficking in hippocampal neurons is highly dynamic, and membrane insertion can occur within minutes (Noam et al., 2010). Impaired regulation of HCN channels is linked to several brain disorders including epilepsy (Albertson et al., 2013; Difrancesco and Difrancesco, 2015) and fragile X mental retardation (Brager et al., 2012; Brandalise et al., 2020). Synaptic plasticity can locally modify the expression and properties of dendritic HCN channels in excitatory neurons (Daoudal and Debanne, 2003; Wang et al., 2003; Shah et al., 2010). Several studies have described that the modulation of HCN channels in pyramidal neurons depends on both the amplitude and direction of synaptic plasticity (van Welie et al., 2004; Fan et al., 2005; Brager and Johnston, 2007; Campanac et al., 2008; Gasselin et al., 2017), thereby contributing to both Hebbian and homeostatic regulation of intrinsic excitability (Debanne et al., 2019). Plasticity rules are often different for excitatory and inhibitory neurons, reflecting their different roles within the local network (Kullmann et al., 2012; Debanne et al., 2019). It is currently unknown if modulation of HCN channels is differentially regulated in inhibitory and excitatory neurons.

Here, we systematically compared the properties of *I*_h_ in hippocampal CA1 pyramidal neurons and stratum radiatum (sRad) interneurons. We quantified changes in *I*_h_ and intrinsic excitability when HCN channels were blocked, following elevated cyclic adenosine monophosphate (cAMP) levels and after synaptic potentiation. Synaptic potentiation was induced by pairing brief bursts of evoked synaptic potentials with postsynaptic depolarization (theta burst pairing) in pyramidal cells and interneurons. Properties of *I*_h_ were found generally similar in excitatory and inhibitory cells. However, while pyramidal cells showed an upregulation of *I*_h_ after strong synaptic potentiation, we found that HCN channels in sRad interneurons were downregulated after synaptic plasticity, irrespective of the strength of the synaptic changes. This suggests that pyramidal cells and interneurons express different mechanisms to modulate *I*_h_, possibly signifying their different roles in the local network.

## Materials and Methods

### Animals

All animal experiments were performed in accordance with the guidelines for the welfare of experimental animals and were approved by the local authorities. Mice were kept in standard cages on a 12 h light/12 h day cycle under SPF conditions. For this study, GAD65-GFP mice (López-Bendito et al., 2004), bred as a heterozygous line with C57BL/6JRj background, or their wild-type littermates, of both sexes, were used. In the hippocampus of GAD65-GFP mice, ∼20% of GABAergic interneurons express GFP. GFP-labeled neurons are mainly reelin and vasoactive intestinal peptide (VIP) positive, while they mostly do not express parvalbumin or somatostatin (Wierenga et al., 2010). These cells also express neuropeptide Y, cholecystokinin, calbindin, or calretinin. Previous studies have shown that, in the mouse hippocampus, *I*_h_ could be recorded in most of these interneuron subtypes (Tricoire et al., 2011; Francavilla et al., 2018).

### Slice preparation

Organotypic hippocampal slice cultures were prepared at postnatal day (P)6–8, as previously described by Stoppini et al. (1991), with some modifications of the protocol. After decapitation, the brain was quickly removed and placed in ice-cold Gey's balanced salt solution [containing the following (in mM): 137 NaCl, 5 KCl, 1.5 CaCl_2_, 1 MgCl_2_, 0.3 MgSO_4_, 0.2 KH_2_PO_4_, 0.85 Na_2_HPO_4_] supplemented with 25 mM glucose, 12.5 mM HEPES, and 1 mM kynurenic acid, with pH 7.2 and osmolarity ∼320 mOsm/l. Both hippocampi were dissected out, and transverse hippocampal slices of 400 μm thick were chopped. The entorhinal cortex (EC) was partially left intact because this area is critical for the development and maintenance of the distal dendritic enrichment of HCN channels in CA1 pyramidal neurons (Shin and Chetkovich, 2007). Slices were placed on Millicell membrane inserts (Millipore Sigma, PICM0RG50) in six-well plates containing 1 ml culture medium (consisting of 48% MEM, 25% HBSS, 25% horse serum, 25 mM glucose, and 12.5 mM HEPES), with pH 7.3–7.4 and osmolarity ∼325 mOsm/l per well. Slice cultures were stored in an incubator (35°C with 5% CO_2_), and the medium was replaced three times a week. Experiments were performed after 10–22 days *in vitro* (DIV).

### Electrophysiology

Before the start of the experiment, a slice was transferred to the recording chamber of the microscope. Artificial cerebral spinal fluid [ACSF; consisting of the following (in mM): 126 NaCl, 3 KCl, 2.5 CaCl_2_, 1.3 MgCl_2_, 26 NaHCO_3_, 1.25 NaH_2_PO_4_, 20 D-glucose, and 1 Trolox, with an osmolarity of 315 ± 10 mOsm/l] was carbonated (95% O_2_, 5% CO_2_), warmed to 30–32°C, and continuously perfused at a speed of ∼1 ml/min. A 4× air objective (Nikon Plan Apochromat) was used to locate the hippocampal CA1 region, and cells were visualized with a 60× 1.0 NA water immersion objective (Nikon NIR Apochromat). Whole-cell patch–clamp recordings were made of CA1 pyramidal neurons and GFP-expressing interneurons. GFP-positive inhibitory cells were identified in the sRad, 100–250 μm from the CA1 pyramidal cell layer, using two-photon fluorescence microscopy. Recording pipettes (resistance of 4–6 MΩ) were filled with an internal solution for measuring excitatory postsynaptic currents (EPSCs; in mM: 140 K-gluconate, 4 KCl, 0.5 EGTA, 10 HEPES, 4 MgATP, 0.4 NaGTP, 4 Na_2_-phosphocreatine), with pH 7.3 and osmolarity 295 ± 5 mOsm/l, or high chloride internal solution to measure inhibitory postsynaptic currents (IPSCs; in mM: 70 K-gluconate, 70 KCl, 0.5 EGTA, 10 HEPES, 4 MgATP, 0.4 NaGTP, 4 Na_2_-phosphocreatine), with pH 7.3 and osmolarity 295 ± 5 mOsm/l. Inhibitory currents were isolated by addition of DL-AP5 (50 μM, Tocris Bioscience) and DNQX (20 μM, Tocris Bioscience) to the ACSF. Spontaneous action potential (AP) firing was prevented by adding 1 μM TTX (Abcam) for the recording of miniature IPSCs. For wash-in experiments, ACSF was substituted with 10 µM ZD7288 (ZD, Sigma-Aldrich) or 25 µM forskolin (FSK, Abcam). During all experiments, cells were kept at a holding potential of −60 mV in both voltage and current clamp. Recordings were excluded when the initial resting membrane potential was above −50 mV. Only interneurons with a *V*_sag_ >5 mV at −300 pA current injection were included (this cutoff was empirically chosen; *V*_sag_ at −400 pA was >5 mV for all pyramidal cells). Recordings were acquired using a MultiClamp 700B amplifier (Molecular Devices) with the pClamp 10 software.

### Electrical stimulation

To visualize the dendritic arbor after patching, 30 µM Alexa Fluor 568 was added to the internal solution (Thermo Fisher Scientific). A concentric bipolar stimulation electrode was placed in a glass pipette filled with ACSF and located in the sRad ∼100–150 μm from the soma of the patched pyramidal neuron. For interneurons, we placed the stimulation pipette in close proximity to a dendrite 50–100 μm from the soma of the patched cell. For stimulation experiments in excitatory neurons, the CA1 area was surgically isolated to prevent recurrent activity, by a cut separating the CA1 and CA3 region and a cut between the CA1 and the EC. Theta burst stimulation (TBS) was considered successful if synaptic responses were enhanced right after the stimulation. Synaptic potentiation was determined as the average responses 30 or 60 min after TBS, normalized to the average response before stimulation (%EPSC). We discriminated between moderate (<150%) and strong (>150%) potentiation (Campanac et al., 2008). Synaptic responses in inhibitory neurons were consistent over time, but extracellular stimulation in pyramidal neurons often evoked variable and multisynaptic responses that could not be avoided by varying the electrode location or stimulation strength. This may reflect an increased connectivity in slice cultures compared with acute slices. In many pyramidal cells, the large variability in the responses made it impossible to unambiguously determine the synaptic potentiation strength after TBS. Experiments in which the extracellular electrode directly stimulated the recorded neuron were disregarded.

For baseline recordings, the stimulus intensity and duration were adjusted to evoke subthreshold postsynaptic currents (evoked PSCs) recorded at 0.1 Hz. We did not block inhibitory currents in these experiments, as washing in GABA_A_ receptor antagonists (bicuculline or gabazine) resulted in massive excitatory currents and cells escaping voltage clamp already at low stimulus strengths, making it impossible to record subthreshold evoked PSCs. Synaptic potentiation was induced after a 10 min stable baseline with a TBS paired with postsynaptic depolarization. Theta-modulated burst firing is a behaviorally relevant activity pattern and more efficient in inducing long-term potentiation than other stimulation protocols (Larson and Munkácsy, 2015). One episode of TBS contained 10 bursts at 5 Hz (200 ms intervals) with each burst consisting of five pulses at 100 Hz. One to three TBS episodes were given at 10 s intervals. Evoked PSCs were paired with backpropagating APs elicited by direct somatic current injection (2 ms, 1 nA; with a 5 ms delay). We included three pyramidal cells in which TBS was not paired with postsynaptic depolarization. We continued to record evoked PSCs for at least 30 min after the TBS protocol; every 2 min, we evoked three responses with an interval of 10 s. In between the evoked PSC recordings, we acquired current stimulation recordings.

### Immunochemistry and confocal imaging

Organotypic hippocampal slices were stained for HCN1 to assess the dendritic HCN channel distribution. Slices were fixed in 4% paraformaldehyde for 30 min at room temperature. Next, slices were permeabilized with 0.5% Triton X-100 for 15 min and incubated for 1 h in blocking solution (0.2% Triton X-100 and 10% goat serum). Primary antibodies mouse α-HCN1 (1:1,000; NeuroMab, N70/28) and chicken α-MAP2 (1:5,000; Abcam/Bio-Connect, ab5392) in blocking solution were applied for 24 h at 4°C. Following extensive washing, slices were incubated at room temperature for 3–4 h with secondary antibody mixture containing Alexa Fluor 568 anti-mouse (1:500; Thermo Fisher Scientific, A11031) and Alexa Fluor 647 anti-chicken (1:500; Thermo Fisher Scientific, A21449). Slices were mounted with Vectashield medium (Vector Laboratories). Confocal laser scanning microscopy images of these slices were acquired on a Zeiss LSM-700 system with a Plan Apochromat 20× 0.8 NA objective. *Z*-stack images were acquired with a step size of 1 μm at 1.6 pixels/µm and tiled to construct an image of the whole slice.

### Data analysis

Electrophysiological data were analyzed with the Clampfit 10.7 software and custom-written MATLAB scripts. From negative current injection recordings, we determined the negative peak as the maximum voltage (*V*_max_), and the total hyperpolarization reached at the end of the current injection as steady-state voltage (*V*_ss_), from which the *V*_sag = _*V*_max _− *V*_ss_ was determined. To assess the activation of *I*_h_, we recorded a two-step protocol, with the first step ranging from −50 to −120 mV (with 10 mV intervals), followed by a second step to −60 mV. Amplitudes of the tail currents (*I*_tail_) were normalized to the maximum *I*_tail_ and plotted versus the membrane potential. *I*_h_ activation curves for each cell were fitted with a Boltzmann function, $I(V)=A_{2}+\frac{A_{1}-A_{2}}{1+e^{\left(\frac{V_{50}-V}{s}\right)}\;}\;$, where *V* is the step voltage, *V*_50_ is the half-activation voltage, *s* is the slope factor, and *A*_1_ and *A*_2_ represent the upper and lower *I*_tail_ amplitudes. Normalized *I*_tail_ amplitudes were averaged to show the mean *I*_h_ activation curve which was refitted by the Boltzmann equation with *A*_2_ set to 0 and *A*_1_ set to 1.

To characterize AP firing properties of the recorded neurons, we determined the number of APs for each 500 ms current injection. The number of APs around threshold in Figure 3, *G*, *H*, and *J*, was determined as the number of APs fired at the smallest current injection step (approximately rheobase). The latency of the first AP was determined at rheobase, except in Figure 4K, which shows the latency of the first AP for all current steps.

### Statistical analysis

Statistical analysis was performed with Prism 9 (GraphPad). Shapiro–Wilk tests were used to test normality. For normally distributed data points, the statistical significance for paired or unpaired samples was evaluated using the paired or unpaired Student's *t* test (*t* test), respectively. For non-normal distributed unpaired or paired data, we used the nonparametric Mann–Whitney (MW) test or Wilcoxon (W) signed-rank test, respectively. An ANOVA was used to compare normally distributed datasets with multiple measurements with the Friedman test as nonparametric alternative. Kolmogorov–Smirnov (KS) tests were used to compare cumulative distributions. *p* < 0.05 was considered significant. All data are presented as mean ± SEM. Details of the statistical tests are provided in Table 1.

## Results

### *I*_h_ contributes to membrane properties in excitatory and inhibitory neurons

**Figure 1.**
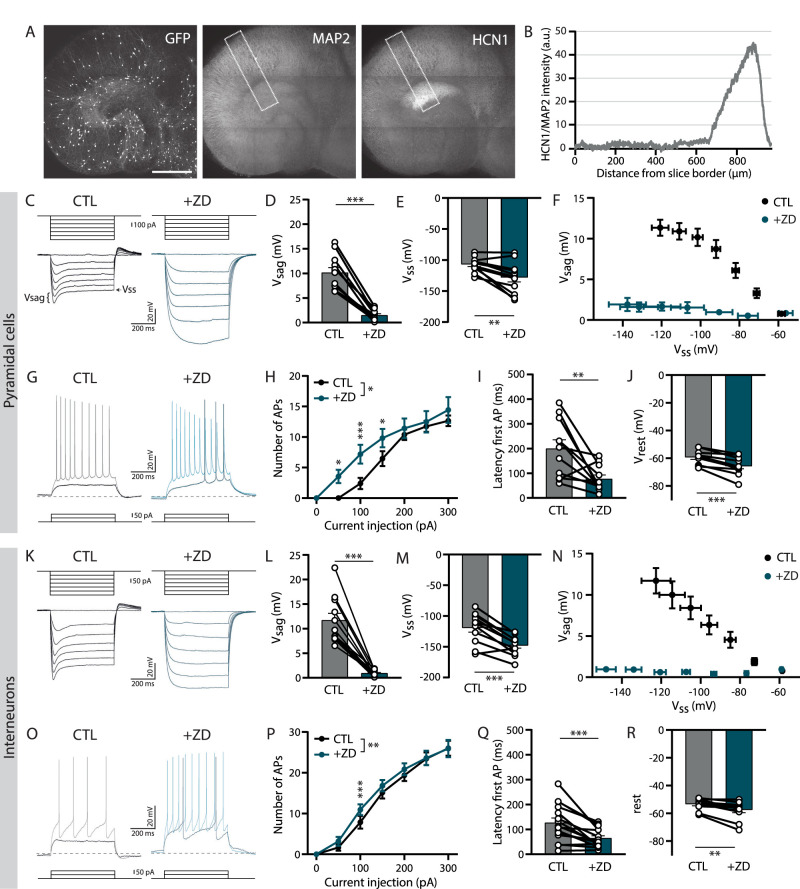
Membrane properties of excitatory and inhibitory neurons are altered by *I*_h_ blockade. ***A***, Immunostaining for HCN1 and MAP2 in an organotypic hippocampal slice from a GAD65-GFP mouse at 18 DIV. Similar antibody labeling was observed in five slices of two mice. Scale bar, 500 µm. ***B***, Quantification of the immunostaining in ***A***. HCN1 normalized to MAP2 intensity, plotted over the distance from the slice border in the area indicated by the rectangle in ***A***. ***C***, *I*_h_ recordings in a pyramidal cell with current injections from 0 to −600 pA (with steps of 100 pA) during the baseline control (CTL; black) and after wash-in of ZD7288 (+ZD; blue). Voltage sag (*V*_sag_) and steady-state voltage (*V*_ss_) are indicated. ***D***, Voltage sag (*V*_sag_) measured in pyramidal cells at −400 pA current injection (*n* = 11; *p* < 0.0001; *t* test). ***E***, Steady-state voltage (*V*_ss_) measured in pyramidal cells at −400 pA current injection (*n* = 11; *p* = 0.005; *t* test). ***F***, Relationship between voltage sag (*V*_sag_) and steady-state voltage (*V*_ss_) measured in pyramidal neurons (*n* = 11; *V*_sag_, *p* < 0.0001; *V*_ss_, *p* = 0.2 two-way ANOVA). ***G***, AP recordings in a pyramidal cell for 50 pA (CTL, black; +ZD, blue) and 100 pA (CTL: gray; +ZD, light blue) current injections. Dotted line indicates the holding potential −60 mV. ***H***, Average number of APs fired in pyramidal neurons for all current injections (*n* = 11; *p* = 0.048 two-way ANOVA with multiple comparisons at 50 pA, *p* = 0.02; 100 pA, *p* = 0.0006; and 150 pA, *p* = 0.03). ***I***, Latency of the first AP in pyramidal neurons at the smallest current injection in CTL (*n* = 11; *p* = 0.008; *t* test). ***J***, Resting membrane potential (*V*_rest_) in pyramidal neurons (*n* = 11; *p* < 0.0001; *t* test). ***K***, I_h_ recordings in an interneuron for current injections from 0 to −300 pA (with steps of 50 pA) during baseline (CTL; black) and wash-in of ZD7288 (+ZD; blue). ***L***, Voltage sag (V_sag_) measured in interneurons at −300 pA current injection (*n* = 11, *p* < 0.0001, *t* test). ***M***, Steady-state voltage (V_ss_) measured in interneurons at −300 pA current injection (*n* = 11, *p* = 0.0001, *t* test). ***N***, Relationship between voltage sag (V_sag_) and steady-state voltage (V_ss_) in interneurons (*n* = 11, V_sag_: *p* < 0.0001, V_ss_: *p* < 0.0001, two-way ANOVA). ***O***, AP recordings in an interneuron for 50 pA (CTL: black, +ZD: blue) and 100 pA (CTL: gray, +ZD: light blue) current injections. Dotted line indicates the holding potential −60 mV. ***P***, Average number of APs fired for current injections from 0 to 300 pA in interneurons (*n* = 15; *p* = 0.009; two-way ANOVA). ***Q***, Latency of the first AP in interneurons at the smallest current injection in CTL (*n* = 15; *p* = 0.0005; *t* test). ***R***, Resting membrane potential (*V*_rest_) in interneurons (*n* = 15; *p* = 0.002; *t* test).

In the hippocampal CA1 area, the density of HCN channels on pyramidal neuron dendrites follows a gradient with higher HCN channel density on distal compared with proximal dendrites (Magee, 1998; Lörincz et al., 2002; Harnett et al., 2015). This density gradient of HCN channels efficiently counteracts dendritic filtering (Vaidya and Johnston, 2013) and depends on synaptic activity from entorhinal inputs (Shin and Chetkovich, 2007). Of the four HCN isoforms, HCN1 is highly enriched in the hippocampus (Monteggia et al., 2000; Dougherty et al., 2013). By using antibody staining for HCN1, we confirmed that the HCN channel density gradient is maintained in our organotypic slice cultures (Fig. 1*A*,*B*).

We performed whole-cell patch–clamp recordings in CA1 pyramidal cells and observed a clear activating *I*_h_ upon negative current injections in all cells, resulting in the so-called voltage sag (*V*_sag_; Fig. 1*C*). The *V*_sag_ was completely abolished when we blocked HCN channels using the selective blocker ZD7288 (ZD; Gasparini and DiFrancesco, 1997; Fig. 1*D*). ZD also resulted in an increase of the steady-state voltage (*V*_ss_) that was reached during the current injections (Fig. 1*E*). The decrease in *V*_sag_ and increase in *V*_ss_ due to the loss of *I*_h_ were directly related (Fig. 1*F*). When *I*_h_ was blocked, we also observed an increase in intrinsic excitability in pyramidal neurons, determined by AP firing (Fig. 1*G*). The increase in the number of APs was specific for smaller current injections (Fig. 1*H*). Consistently, the latency of the first AP was also decreased upon ZD application (Fig. 1*I*). In addition, we observed a significant hyperpolarization of the resting membrane potential (*V*_rest_) after adding ZD (Fig. 1*J*). These observations were in good agreement with previous reports (Maccaferri and McBain, 1996; Magee, 1999; Poolos et al., 2002; Fan et al., 2005; Tokay et al., 2009). This shows that *I*_h_ properties of CA1 pyramidal neurons in our slice cultures were similar to acute slices.

Next, we recorded from GFP-labeled sRad interneurons in slices from GAD65-GFP mice. These interneurons are mostly reelin-positive and target pyramidal cell dendrites (Wierenga et al., 2010). We could measure *I*_h_ in ∼70% of the GFP-expressing sRad interneurons (Fig. 1*K*). As in pyramidal cells, blocking HCN channels with ZD consistently resulted in an elimination of the *V*_sag_ and an increase in the *V*_ss_ (Fig. 1*L*–*N*). Application of ZD also affected AP firing at small current injections in sRad interneurons (Fig. 1*O*–*Q*), although this effect was diluted due to large variability in firing threshold between the interneurons. *I*_h_ blockade hyperpolarized *V*_rest_ (Fig. 1*R*). Our results suggest that *I*_h_ similarly affects membrane properties and AP firing in CA1 pyramidal cells and sRad interneurons.

### Increased cAMP levels shift the activation curve of *I*_h_ in excitatory and inhibitory neurons

**Figure 2.**
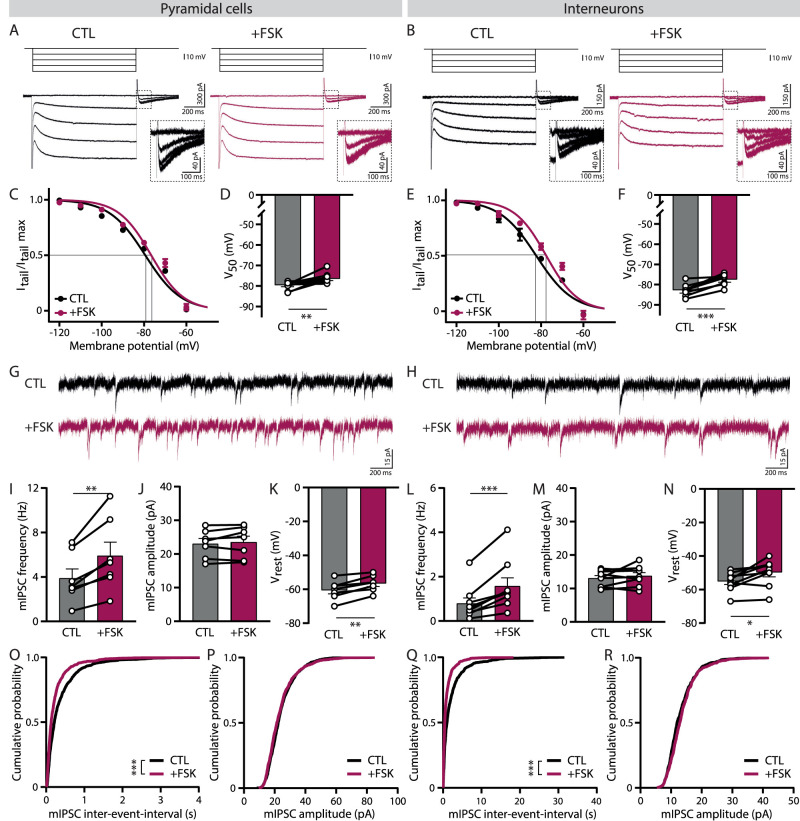
Increase in cAMP with FSK shifts the activation curve of *I*_h_ in excitatory and inhibitory neurons. ***A***, *I*_h_ recordings in a pyramidal cell for voltage steps from −60 to −100 mV (with steps of 10 mV) during baseline (CTL; black) and wash-in of FSK (+FSK; purple). Insert shows a zoom of the tail currents measured at −60 mV. ***B***, *I*_h_ recordings in an interneuron for voltage steps from −60 to −100 mV (with steps of 10 mV) during baseline (CTL; black) and wash-in of FSK (+FSK; purple). Insert shows a zoom of the tail currents measured at −60 mV. ***C***, Average activation curve of *I*_h_ in pyramidal cells constructed from normalized tail currents (*I*_tail_ / *I*_tail_ max) versus membrane potentials (*n* = 10). ***D***, For each cell, activation curves were fitted with a Boltzmann function. *V*_50_ values for pyramidal cells in CTL and FSK conditions (*n* = 10, *p* = 0.01, W test). ***E***, Average activation curve of *I*_h_ in interneurons constructed from normalized tail currents (I_tail_ / I_tail_ max) versus membrane potentials (*n* = 8). ***F***, For each cell, activation curves were fitted with a Boltzmann function. *V*_50_ values for interneurons in CTL and FSK conditions (*n* = 8; *p* = 0.0004; *t* test). ***G***, Example of mIPSCs recorded in a pyramidal during baseline (CTL; black) and wash-in of FSK (+FSK; purple). ***H***, Example of mIPSCs recorded in an interneuron during baseline (CTL; black) and wash-in of FSK (+FSK; purple). ***I***, Average miniature IPSC frequency in pyramidal cells (*n* = 7; *p* = 0.004; *t* test). ***J***, Average miniature IPSC amplitude in pyramidal cells (*n* = 7; *p* = 0.36; *t* test). ***K***, Resting membrane potential (*V*_rest_) of pyramidal cells (*n* = 7; *p* = 0.002; *t* test). ***L***, Average miniature IPSC frequency in interneurons (*n* = 9; *p* = 0.001; W test). ***M***, Average miniature IPSC amplitude in interneurons (*n* = 9; *p* = 0.65; *t* test). ***N***, Resting membrane potential (*V*_rest_) of interneurons (*n* = 9; *p* = 0.01; *t* test). ***O***, Cumulative distributions of mIPSC interevent interval measured in pyramidal cells (*n* = 7; *p* < 0.0001; KS test). For each cell, 150 mIPSCs were randomly selected. ***P***, Cumulative distributions of mIPSC amplitude measured in pyramidal cells (*n* = 7; *p* = 0.008; KS test). For each cell, 150 mIPSCs were randomly selected. ***Q***, Cumulative distributions of mIPSC interevent interval measured in interneurons (*n* = 8; *p* < 0.0001; KS test). For each cell, 75 mIPSCs were randomly selected. ***R***, Cumulative distributions of mIPSCs amplitude measured in interneurons (*n* = 8; *p* = 0.007; KS test). For each cell, 75 mIPSCs were randomly selected.

We assessed the activation properties of HCN channels in hippocampal pyramidal neurons and sRad interneurons by measuring tail currents (*I*_tail_) after variable voltage steps. We then constructed an activation curve of *I*_h_ by plotting the amplitude of the *I*_tail_ for each voltage step (Fig. 2*A*,*B*). HCN channels are sensitive to cyclic nucleotides, including cAMP (Biel et al., 2009). cAMP directly binds to HCN channels and shifts the activation curve of *I*_h_ toward less negative potentials (Wainger et al., 2001; Dini et al., 2018). To determine how HCN channel activation is regulated by cAMP in CA1 pyramidal cells and sRad interneurons, we applied FSK, which rapidly elevates intracellular cAMP levels via activation of adenylyl cyclase. We observed a small depolarizing shift in the *I*_h_ activation curve during FSK application in pyramidal cells (Fig. 2*C*). Normalized *I*_tail_ during FSK was smaller for larger voltage steps, resulting in a more depolarized *V*_50_ after cAMP upregulation (Fig. 2*D*). *I*_h_ activation curves in interneurons appeared shifted to slightly more hyperpolarized values compared with pyramidal cells under baseline conditions (*V*_50_ pyramidal cells, −79.6 ± 0.6 mV; *V*_50_ interneurons, −82.8 ± 1.1 mV; *p* = 0.054^a^ MW test), while FSK application resulted in a similar depolarizing shift (Fig. 2*E*,*F*).

We verified that FSK had fully penetrated the slice by recording miniature IPSCs (mIPSCs; Fig. 2*G*,*H*). In pyramidal neurons, the frequency of mIPSCs increased almost twofold within 5 min of FSK wash-in, while mIPSC amplitude was unaffected (Fig. 2*I*,*J*). This reflects the acute elevation of cAMP levels in presynaptic GABAergic terminals by FSK (Kaneko and Takahashi, 2004; Huang and Hsu, 2006). In parallel to the increase in mIPSCs, we observed a significant depolarization of *V*_rest_ in pyramidal cells during FSK wash-in (Fig. 2*K*), which was consistent with the observed change in the voltage dependence of *I*_h_ activation. A similar increase in mIPSC frequency and depolarization of *V*_rest_ was observed in inhibitory neurons (Fig. 2*L*–*N*). Cumulative distributions of mIPSC amplitudes and interevent intervals were consistent (Fig. 2*O*–*R*). We noticed that mIPSC frequency remained elevated or at least 30 min after we stopped washing in FSK, while *V*_rest_ returned to its initial value (data not shown). Together, these data suggest that HCN channels in interneurons and pyramidal cells are comparably sensitive to cAMP and that in both cell types *I*_h_ gets facilitated by elevating cAMP levels.

### Bidirectional modulation of *I*_h_ by synaptic potentiation in excitatory neurons

**Figure 3.**
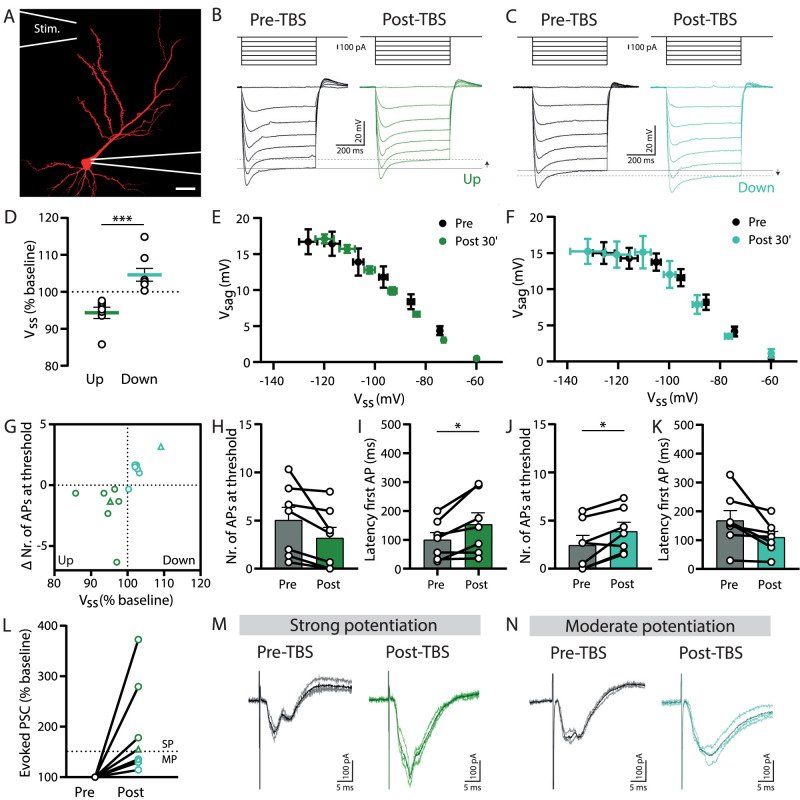
Modulation of *I*_h_ in pyramidal neurons depends on the strength of synaptic potentiation. ***A***, A pyramidal neuron filled with Alexa Fluor 568 via the patch pipette. The location of the stimulation pipette (Stim.) is indicated. Scale bar, 20 µm. ***B***, *I*_h_ recordings for current injections from 0 to −600 pA (with steps of 100 pA) in a pyramidal neuron before (pre-TBS, black) and after TBS (post-TBS, green). Arrow indicates an increase in *V*_ss_, indicating an upregulation of *I*_h_. ***C***, *I*_h_ recordings for current injections from 0 to −600 pA (with steps of 100 pA) in a pyramidal neuron before (pre-TBS, black) and after TBS (post-TBS, green). Arrow indicates a decrease in *V*_ss_, indicating a downregulation of *I*_h_. ***D***, Average change in steady-state voltage (*V*_ss_, % baseline) at −400 pA current injection for experiments in which *I*_h_ showed up- and downregulation (up, *n* = 7; down, *n* = 8; *p* = 0.0003; MW test). ***E***, Relationship between voltage sag (*V*_sag_) and steady-state voltage (*V*_ss_) during the baseline (pre-) and 30 min post-TBS (average of 3 recordings) in pyramidal cells that showed *I*_h_ upregulation (*n* = 6; *V*_sag_, *p* = 0.04,;*V*_ss_, *p* < 0.0001; two-way ANOVA). ***F***, Relationship between voltage sag (*V*_sag_) and steady-state voltage (*V*_ss_) during the baseline (pre-) and 30 min post-TBS (average of 3 recordings) in pyramidal cells that showed *I*_h_ downregulation (*n* = 7; V_sag_, *p* = 0.8; *V*_ss_, *p* = 0.2; two-way ANOVA). ***G***, Correlation between the change in number of APs fired around threshold and the change in steady-state voltage (*V*_ss_, % baseline). Colors indicate data from pyramidal cells with *I*_h_ upregulation (dark green) and *I*_h_ downregulation (light green). Triangles represent the examples shown in ***M*** and ***N***. ***H***, The number of APs fired around threshold during baseline (pre-) and 30 min post-TBS (*n* = 11; *p* = 0.056; *t* test) for pyramidal cells with *I*_h_ upregulation. ***I***, Latency of the first AP fired during the baseline (pre-) and 30 min post-TBS (*n* = 11; *p* = 0.02; *t* test) for pyramidal cells with *I*_h_ upregulation. ***J***, The number of APs fired around threshold during the baseline (pre-) and 30 min post-TBS (*n* = 11; *p* = 0.011; *t* test) for pyramidal cells with *I*_h_ downregulation. ***K***, Latency of the first AP fired during baseline (pre-) and 30 min post-TBS (*n* = 11; *p* = 0.051; *t* test) for pyramidal cells with *I*_h_ downregulation. ***L***, Experiments were categorized in moderate synaptic potentiation (MP, evoked PSC <150% of the baseline 20 min post-TBS; *n* = 4) and strong synaptic potentiation (SP, evoked PSC >150%; *n* = 4). Dashed line indicates 150% potentiation. Triangles represent the examples shown in ***M*** and ***N***. ***M***, Example of strong synaptic potentiation. Evoked PSCs during the baseline (pre-TBS; average, black; individual traces, gray) and after TBS (post-TBS; average, green; individual traces, light green). ***N***, Example of moderate synaptic potentiation. Evoked PSCs during the baseline (pre-TBS; average, black; individual traces, gray) and after TBS (post-TBS; average, green; individual traces, light green).

Plasticity of intrinsic excitability via HCN channels has been well described in excitatory neurons (van Welie et al., 2004; Fan et al., 2005; Campanac et al., 2008). To confirm the modulation of *I*_h_ in CA1 pyramidal neurons in our organotypic slices, we applied TBS paired with postsynaptic depolarization (hereafter referred to as TBS) via an extracellular electrode ∼100–150 µm from the soma to stimulate dendritic synapses (Fig. 3*A*). TBS induced consistent changes in *I*_h_ upon hyperpolarizing current steps. In some pyramidal cells, *V*_ss_ was consistently reduced after TBS, indicative of an upregulation of *I*_h_ (Fig. 3*B*), while in other pyramidal cells *V*_ss_ was increased after TBS, consistent with a downregulation of *I*_h_ (Fig. 3*C*). We therefore separated the experiments in two groups, according to the observed change in *V*_ss_ (Fig. 3*D*). The changes in *V*_ss_ were accompanied by changes in *V*_sag_ (Fig. 3*E*,*F*), in line with either an up- or downregulation of HCN channels after TBS. The change in *V*_ss_ was well correlated with changes in firing properties around threshold (Fig. 3*G*). An upregulation of *I*_h_ was accompanied with a small decrease in the number of APs around threshold and an increase in the latency to the first AP (Fig. 3*H*,*I*). In pyramidal cells in which TBS resulted in a downregulation of *I*_h_, the number of APs was increased, and AP latency was decreased (Fig. 3*J*,*K*). This is consistent with our earlier observations of the contribution of *I*_h_ to AP firing (Fig. 1*H*,*I*). Together these observations indicate that TBS induced HCN channel modulation in pyramidal cells. However, it is important to note here that our data do not exclude additional changes to other ion channels after TBS (Sammari et al., 2022).

Previous studies have demonstrated that the direction of HCN channel modulation depends on the strength of synaptic potentiation after TBS. Moderate synaptic potentiation was mostly accompanied by a downregulation of *I*_h_, while upregulation of *I*_h_ was triggered after strong synaptic potentiation (Fan et al., 2005; Campanac et al., 2008). In our experiments, the extracellular stimulation often evoked highly varying and multisynaptic responses in many pyramidal cells, which prevented unambiguous quantification of the strength of the synaptic potentiation (also see Materials and Methods). Although we could not systematically correlate *I*_h_ changes with synaptic potentiation strength for all experiments, our results were in general agreement with previous studies. Strong synaptic potentiation was observed in pyramidal cells in which *I*_h_ was upregulated after TBS, while downregulation of *I*_h_ was associated with more moderate synaptic potentiation (Fig. 3*L*–*N*). These results indicate a bidirectional modulation of *I*_h_ in CA1 pyramidal neurons after theta burst potentiation of synaptic inputs in organotypic hippocampal slices.

### Downregulation of *I*_h_ by synaptic plasticity in inhibitory neurons independent of the strength of potentiation

**Figure 4.**
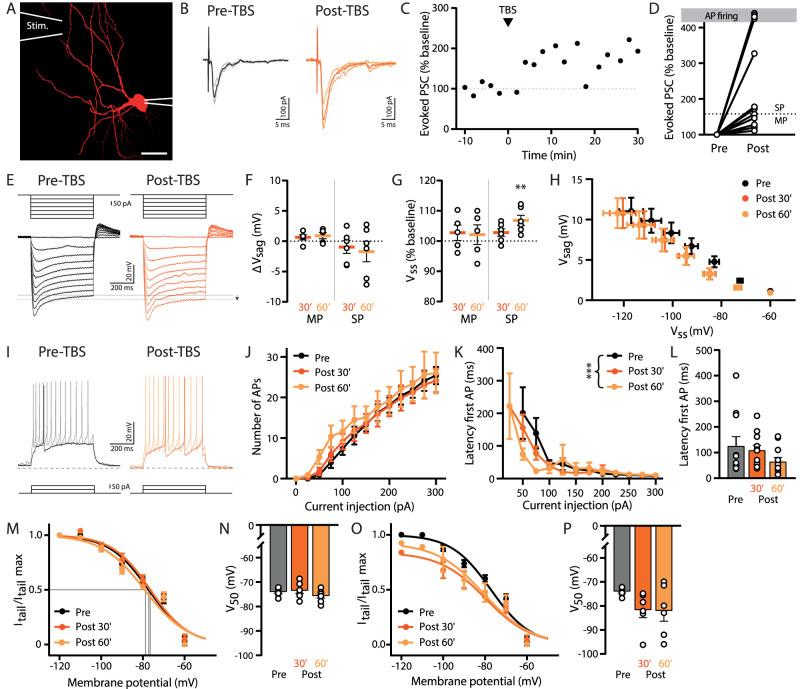
Downregulation of *I*_h_ in interneurons independent of the strength of synaptic potentiation. ***A***, An interneuron filled with Alexa Fluor 568 via the patch pipette. The location of the stimulation (Stim.) pipette is indicated. Scale bar, 20 µm. ***B***, Evoked PSCs during the baseline (pre-TBS; average, black; individual traces, gray) and after TBS (post-TBS; average, orange; individual traces, light orange). ***C***, Evoked PSC amplitude (% baseline) over time for the experiment shown in ***B***. ***D***, Experiments were categorized in moderate synaptic potentiation (MP, evoked PSC <150% of the baseline 0−20 min after the TBS; *n* = 5) and strong synaptic potentiation (SP, evoked PSC >150%; *n* = 6). Dashed line indicates 150% potentiation. ***E***, *I*_h_ recordings in an interneuron for current injections from 0 to −300 pA (with steps of 50 pA) before (pre-TBS, black) and after TBS (post-TBS, orange). Arrow indicates a decrease in *V*_ss_, indicating a downregulation of *I*_h_. ***F***, Voltage sag (Δ*V*_sag_) at −300 pA current injection 30 and 60 min post-TBS (average of 3 recordings) for experiments with moderate and strong synaptic potentiation (MP *n* = 5 and SP *n* = 6). ***G***, Steady-state voltage (*V*_ss,_ % baseline) at −300 pA current injection 30 and 60 min post-TBS (average of 3 recordings) for experiments with moderate and strong synaptic potentiation (MP *n* = 5 and SP *n* = 6; SP 60′, *p* = 0.009; one-sample *t* test). ***H***, Relationship between voltage sag (*V*_sag_) and steady-state voltage (*V*_ss_) during baseline (pre-), 30 and 60 min post-TBS (average of 3 recordings) in interneurons (*n* = 11; *V*_sag_, *p* = 0.5; *V*_ss_, *p* = 0.06; two-way ANOVA). ***I***, AP recordings in an interneuron for 50 pA (pre-TBS, black; post-TBS, orange) and 100 pA (pre-TBS, gray; post-TBS, light orange) current injections. Dotted line indicates −60 mV. ***J***, The average number of APs fired in interneurons for all current injections (*n* = 11; *p* = 0.9; two-way ANOVA). ***K***, Latency of the first AP fired in interneurons for all current injections during baseline (pre-), 30 and 60 min post-TBS (*n* = 11; *p* < 0.0001 mixed-effect model). ***L***, Latency of the first AP in interneurons at the smallest current injection pre-TBS (*n* = 11; *p* = 0.1; Friedman test). ***M***, Average *I*_h_ activation curve constructed by normalizing tail currents to the maximum *I*_tail_ per condition (*n* = 7). Curves were fitted with Boltzmann functions. ***N***, *V*_50_ values determined from individual *I*_h_ activation curves as shown in ***N*** (*n* = 7; *p* = 0.3; one-way ANOVA). ***O***, Average *I*_h_ activation curve constructed by normalizing tail currents (*I*_tail_) to the maximum *I*_tail_ during the baseline (pre-TBS, *n* = 7). Curves were fitted with Boltzmann functions. ***P***, *V*_50_ values determined from individual *I*_h_ activation curves as shown in ***O*** (*n* = 7; *p* = 0.1; one-way ANOVA).

Next we recorded from GFP-positive interneurons in the sRad of the hippocampal CA1 region (Fig. 4*A*). We only included inhibitory neurons in which *I*_h_ could be recorded (∼75% of patched cells). We recorded evoked PSCs for 10 min (baseline) and then induced synaptic potentiation with TBS paired with postsynaptic depolarization (Fig. 4*B*). Evoked PSCs were recorded for at least 30 min after TBS (Fig. 4*C*). Most sRad interneurons showed moderate to strong synaptic potentiation, and in a few cells (4 out of 11), the extracellular stimulation evoked AP firing after TBS (Fig. 4*D*). We recorded the change in membrane potential for incrementing negative current injections before and after TBS to quantify *I*_h_ (Fig. 4*E*). We did not observe a significant change in *V*_sag_ or *V*_ss_ 30 min after TBS, but after 60 min, the *V*_ss_ was more hyperpolarized (Fig. 4*F*–*H*). This was particularly clear in interneurons that had undergone strong synaptic potentiation. All experiments combined, the average *V*_ss_ at −300 pA current injection was significantly different from the baseline after 60 min (*p* = 0.03^b^; one-sample *t* test). We also observed a small increase in AP firing and decrease in latency of the first AP upon small current injections (≤100 pA) 60 min after synaptic potentiation (Fig. 4*I*–*L*). The activation curve of *I*_h_ did not change after synaptic potentiation when *I*_tail_ was normalized to their own maximum (Fig. 4*M*,*N*), indicating that *I*_h_ activation kinetics were not affected. When we normalized *I*_tail_ to the maximum *I*_tail_ in the baseline, the reduction in maximum *I*_tail_ after synaptic potentiation was clearly noticeable (Fig. 4*O*,*P*). This shows that TBS-induced synaptic potentiation in sRad interneurons results in a downregulation of *I*_h_ without affecting HCN channel kinetics.

## Discussion

In this study, we compared *I*_h_ in pyramidal cells and sRad interneurons in the hippocampal CA1 area. We found that *I*_h_ properties were similar and that *I*_h_ contributes significantly to *V*_rest_ and AP firing in both types of neurons. TBS induced synaptic potentiation in both cell types, which was associated with either an up- or downregulation of *I*_h_ in pyramidal cells, while TBS only induced downregulation of *I*_h_ in sRad interneurons. Our data indicate that HCN channel regulation is cell-type specific, and we speculate that this may signify the different roles of these neurons in the local network.

The density gradient and recordings of *I*_h_ (i.e., *V*_sag_ and *V*_ss_) in CA1 pyramidal cells in our experiments were similar to previous reports in acute slices (Magee, 1998; Wilkars et al., 2012; Srinivas et al., 2017). In CA1 pyramidal cells, dendritic HCN channels are distributed following a density gradient with higher densities further away from the soma (Lörincz et al., 2002; Harnett et al., 2015). The cellular mechanism underlying the dendritic gradient of *I*_h_ remains unclear (Kupferman et al., 2014; Meseke et al., 2018), but it was shown to require intact projections from the EC (Shin and Chetkovich, 2007). We keep a part of the EC attached to our hippocampal slices to maintain the CA1 network architecture (Brewster et al., 2006; Shin and Chetkovich, 2007). After 2 weeks in culture, our hippocampal slices would be roughly equivalent to the third postnatal week in vivo (De Simoni et al., 2003)*.* It is less clear if HCN channels also have a specific cellular distribution in interneurons. In parvalbumin cells, HCN channels are highly enriched in axons (Aponte et al., 2006; Elgueta et al., 2015; Roth and Hu, 2020), but in other interneurons, HCN channels appear to be localized mostly in the soma and proximal dendrites (Anderson et al., 2011; Sekulić et al., 2015, 2020).

From our experiments, there are no indications that the properties of HCN channels are different in inhibitory and excitatory neurons. We found that *I*_h_ affects passive membrane properties, *V*_rest­_, and input resistance similarly in hippocampal CA1 pyramidal cells and sRad interneurons. *I*_h_ dampens intrinsic excitability, and blocking HCN channels with ZD led to a small increase in AP firing and decrease in *V*_rest_. FSK application had two independent effects: it triggered an increase in mIPSC frequency and shifted the *I*_h_ activation curve in both cell types. FSK activates the enzyme adenylyl cyclase, which converts ATP to the second messenger cAMP. cAMP is known to stimulate presynaptic vesicle release via activation of protein kinases (Weisskopf et al., 1994; Nicoll and Schmitz, 2005; Antoni, 2012). The FSK-mediated increase in mIPSC frequency was similar in pyramidal cells and interneurons and likely reflects a general increase in vesicle release in presynaptic inhibitory terminals in the brain slice (Kaneko and Takahashi, 2004; Huang and Hsu, 2006). In parallel, we observed a depolarizing shift in *I*_h_ activation in both cell types, consistent with previous reports that cAMP can directly influence the gating properties of HCN channels (Wainger et al., 2001; Magee et al., 2015; Dini et al., 2018). Due to the depolarizing shift in gating, more HCN channels are open at rest resulting in a small, but significant, depolarization of *V*_rest_ (Gambardella et al., 2012). We noticed that the *V*_rest_ depolarization was abolished when FSK application ended, while mIPSC frequency remained elevated. This is consistent with the notion that mIPSC increase is mediated via cAMP-dependent kinases (Capogna et al., 1995; Fernandes et al., 2015), while the effect on HCN channels and *V*_rest_ is directly mediated by cAMP (Lüthi and McCormick, 1999).

The most important observation in this study was that synaptic potentiation had a differential effect on HCN channels in pyramidal cells and sRad interneurons. The changes in *I*_h_ were clear from changes in *V*_sag_ and *V*_ss_ during hyperpolarizing current steps. However, it is important to note here that we did not perform pharmacological controls, and we therefore cannot exclude additional changes to other ion channels after TBS (Sammari et al., 2022). Previous studies showed that modulation of HCN channels in hippocampal pyramidal neurons depends on the strength of synaptic potentiation. Large synaptic potentiation causes an upregulation of *I*_h_ and therefore a decrease in intrinsic excitability, whereas moderate potentiation results in a downregulation of *I*_h_ associated with increased intrinsic excitability (van Welie et al., 2004; Fan et al., 2005; Campanac et al., 2008; Debanne et al., 2019). We used TBS to induce synaptic potentiation in pyramidal cells and interneurons. We observed an increase in *I*_h_ only in some pyramidal cells, while in others *I*_h_ was downregulated after TBS. Although we could not directly relate this to the strength of synaptic potentiation in our experiments, our results clearly demonstrate bidirectional *I*_h_ modulation in CA1 pyramidal cells, in line with these previous studies. In contrast, we always observed a reduction in *I*_h_ in sRad interneurons after synaptic potentiation. The difference in *I*_h_ modulation between pyramidal cells and interneurons cannot be explained by a reduced efficiency of synaptic potentiation in the sRad interneurons. In fact, synaptic stimulation resulted in AP firing in some interneurons after TBS, indicating that maximal synaptic potentiation was reached. Our data suggest that inhibitory neurons only exhibit a mechanism to downregulate HCN channels and lack a mechanism for *I*_h_ upregulation.

Previous studies have identified several molecular mechanisms that regulate surface expression and activation properties of HCN channels. The gating properties of *I*_h_ are modulated by cAMP and cGMP (Wainger et al., 2001; Maroso et al., 2016) but also by PIP_2_ (Pian et al., 2006; Zolles et al., 2006). In addition, HCN proteins contain multiple phosphorylation sites by which the number and properties of functional HCN channels in the membrane are regulated (Williams et al., 2015; Concepcion et al., 2021). TBS can increase intracellular cAMP levels (Nguyen and Kandel, 1997; Lüthi and McCormick, 1999), and an increase in cAMP enhances *I*_h_ (Lüthi and McCormick, 1999). We found that HCN channels in excitatory and inhibitory cells are equally sensitive to changes in cAMP levels upon FSK application. The synaptic potentiation-driven reduction in *I*_h_ conductance in interneurons was not accompanied by a change in activation kinetics (Fig. 4*M*–*P*), which suggests that TBS did not elevate cAMP levels in these interneurons. Previous studies have reported downregulation of *I*_h_ also in excitatory neurons, when synaptic potentiation after TBS was moderate. In that case, the reduction in *I*_h_ conductance also occurred without a change in the activation curve (Campanac et al., 2008), very similar to what we observe in sRad interneurons. This suggests that an increase in cAMP may be required for *I*_h_ upregulation after strong synaptic potentiation in pyramidal cells but that moderate synaptic potentiation does not affect cAMP levels. The observed difference in *I*_h_ modulation after TBS stimulation in pyramidal cells and interneurons may therefore suggest a differential effect of the synaptic stimulation on intracellular cAMP levels. In addition, baseline cAMP levels in CA1 pyramidal cells and sRad interneurons may already be different as suggested by the apparent difference in *V*_50_ values of the *I*_h_ activation curve in these cells (Fig. 2*C*,*E*). Future studies could employ novel PKA sensors (Ma et al., 2018) to directly compare cAMP dynamics in excitatory and inhibitory cells after synaptic potentiation. In addition to cAMP, upregulation of *I*_h_ after synaptic potentiation may involve CaMKII and NMDA receptor activation (van Welie et al., 2004; Fan et al., 2005). It is also possible that the absence of *I*_h_ upregulation in sRad interneurons is due to the cell-type–specific expression of some molecular components of the upregulation pathway (e.g., expression of CaMKIIα is much lower in interneurons; Liu and Jones, 1996; Sík et al., 1998; Keaveney et al., 2020; Veres et al., 2023).

Downregulation of *I*_h_ can be mediated via PKC activation (Brager and Johnston, 2007; Williams et al., 2015) or via PLC-mediated depletion of PIP_2_ (Pian et al., 2006; Zolles et al., 2006). A recent report described that downregulation of HCN channels after synaptic potentiation in OLM cells is mediated by mGluR1 activation (Sammari et al., 2022). The downregulation of *I*_h_ and increase in AP firing that we observed here are very similar to what was reported in OLM cells, and it is therefore tempting to speculate that a similar pathway is involved in sRad interneurons. We noticed that the increase in *V*_ss_ in sRad interneurons became significant only after 60 min, suggesting that *I*_h_ downregulation is slower in inhibitory neurons compared with excitatory neurons (Campanac et al., 2008) and OLM cells (Sammari et al., 2022).

Intrinsic excitability and firing properties are highly cell-type specific, reflecting specific genetic programs within cell types, which will be influenced by network activity patterns. In addition, cell-type–specific regulation of ion channels may depend on the role of the neuron in the network. Within neuronal networks, plasticity of excitation is usually accompanied by plasticity of inhibition to ensure fidelity of information processing and to enable computational flexibility (Carvalho and Buonomano, 2009; Froemke, 2015; Herstel and Wierenga, 2021). For both excitation and inhibition, plasticity of synaptic connections and intrinsic excitability are coordinated to achieve changes in network function (Kullmann et al., 2012; Gao et al., 2017; Debanne et al., 2019). Activity- and context-dependent recruitment of inhibitory cells is important for information processing in neuronal networks and behavioral flexibility. It is therefore not surprising that different plasticity rules apply for feedforward and feedback inhibition (Lamsa et al., 2005, 2007; Sambandan et al., 2010), reflecting their different role in the network. The GFP-expressing sRad interneurons in slices from GAD65-GFP mice consist of a broad population of different interneurons subtypes, but they mostly target the dendrites of CA1 pyramidal cells (Wierenga et al., 2010) and provide feedforward inhibition to CA1 pyramidal cells (Cope et al., 2002; Wierenga and Wadman, 2003; Lamsa et al., 2005; Milstein et al., 2015). The upregulation of *I*_h_ in pyramidal cells after strong synaptic potentiation is thought to restrict uncontrolled activity (Debanne et al., 2019). Here we found that sRad interneurons respond to synaptic plasticity by decreasing *I*_h_, which will increase their excitability. An increase in interneuron excitability after strong network activity makes sense from a network point of view. We speculate that the increase in excitability of sRad interneurons is important to strengthen feedforward inhibition and helps to sharpen activity patterns (Lamsa et al., 2005; Kullmann et al., 2012). Plasticity of HCN channels provides an intracellular mechanism to adjust and finely regulate neuronal excitability in reaction to synaptic stimulation. Our data highlight that regulation mechanisms for HCN channels vary between neuronal cell types.

## Synthesis

Reviewing Editor: Arvind Kumar, KTH Royal Institute of Technology Decisions are customarily a result of the Reviewing Editor and the peer reviewers coming together and discussing their recommendations until a consensus is reached. When revisions are invited, a fact-based synthesis statement explaining their decision and outlining what is needed to prepare a revision will be listed below. The following reviewer(s) agreed to reveal their identity: Simonas Griesius.

Synthesis

In this paper authors have characterized the plasticity of HCN channels in hippocampal neurons. Both reviewers think that the manuscript provides new data and potentially provides new insights into the function of HCN channels. While data is of high quality, reviewers note that in some cases reported change is rather small and/or number of cells are too few. Moreover, reviewers think that the reporting of the statistics is not complete and they have made clear suggestions of what they would like to see in the revised version. Other than that, there are some minor issues that should also be addressed in the revised manuscript.

Reviewer #1

This is a nice paper examining changes in HCN function in both pyramidal neurons and interneurons located in the startum radiatum of the CA1 area following induction of LTP. The authors first show that moderate LTP is associated with an increase in input resistance attributable to a reduction in HCN function whereas strong LTP is associated with a reduction in input resistance due to a gain of HCN function. Regarding GABAergic interneurons, they show that HCN channels were downregulated after synaptic plasticity, irrespective of the strength of synaptic potentiation. They conclude that this differential regulation of HCN channels in excitatory and inhibitory neurons possibly signifies their distinct role in network activity.

The data are of high quality and the paper is well written. The paper shows interesting and new results concerning HCN modulation in interneurons located in the stratum radiatum. Nevertheless, I have a few relatively minor points that need to be addressed.

1. Although the results confirm previously published data, the number of pyramidal cells is too low (n=3 and n = 2) to provide significant results. Please increase the number of cells.

2. The delay in the downregulation of HCN channels is intriguing in GABAergic interneurons. This may indicate that it involves a complex signaling cascade and possibly the turnover of HCN channels. Which glutamate receptor is involved in this delayed downregulation?

Reviewer #2

This paper characterises HCN function is s. radiatum interneurons in mouse organotypic hippocampal slices. The findings are compared against HCN function in pyramidal neurons in the same preparation. In addition to investigating intrinsic electrophysiological properties in these two cell types the authors investigate HCN modulation by cAMP using within-subject pharmacology. The authors also investigate HCN modulation by theta burst induced plasticity in both cell types. It is a straightforward and well-written paper with largely robust analysis (some relatively minor issues discussed below). I think the paper advances the field but that the interpretation of the results may benefit from some tempering. Some of the changes reported are small and tenuous, particularly in the LTP experiments. Further, given that there was not a pharmacological control in the LTP experiments, the language in the manuscript should be cautious.

In the methods subsection on electrophysiology it states that Vsag larger than 5 mV at -400 to -300 pA was used. Was there an a priori hypothesis for the cut off? Why 5 mV?

The stimulation electrodes appear to have been placed relatively close to the target cells. Are the authors confident they did not stimulate the cells directly?

Fig 1 O-Q: Example trace suggests increased spiking after ZD, but P value does not robustly support this (ANOVA indicates trend). It could be more appropriate to show a more representative example and edit the wording in the results section. In the last results paragraph discussing fig 1 a correlation is referenced but the data is not shown. Please consider reporting the P and r^2.

Fig 2 G-H: Please show example traces. It could be more appropriate to plot cumulative probabilities for the mIPSC measurements and to use the Kolmogorov-Smirnov test probe differences in distribution.

Fig 3: Is it possible to split the data into 30' and 60' subgroups as in fig 4 to make the data easier to compare across cell types? The authors could consider adding a panel to fig 3 similar to the fig 4 panel H, again for easier comparisons across cell type. Please consider showing responses over time as in fig 4 panel C. Consider showing the group responses in addition to the representative example. Example traces in panel C appear to be polysynaptic. Is this why there was more LTP in this cell? Was the other "strong potentiation" cell also polysynaptic? It wasn't made explicitly clear what constituted an amount of LTP as "moderate" versus "strong" as far as I could tell. How was the 150% cut off chosen? There appear to be 3 data points in the moderate group and 2 data points in the strong group, with an argument in the paper that strong potentiation leads to increased Ih. Generally, it is difficult to be confident in the results at this power. Statistically, there is no change in Vsag and a 5% change in Vss. Might this mean that HCN conductance is unchanged (since there was no change in Vsag) and that another channel is responsible for changes in Vss? A pharmacological control could be useful as in Fan et al. 2005, cited in the discussion. The authors could also test for a correlation between evoked PSC % baseline and Vsag and Vss using the data in the figure to strengthen their argument. In the final sentence of the results paragraph on fig 3 it states "...results confirm the bidirectional..." but I think the evidence for this is relatively weak. In panels G and H, there is either no change in the moderate potentiation group or a small tentative change in the strong potentiation group. I recommend rewording the interpretation to be more cautious.

Fig 4: The authors could consider showing the group responses in addition to the representative example. There is a sentence in the results section discussing fig 4 "This was particularly clear in interneurons that had undergone strong synaptic potentiation." The authors may wish to strengthen this statement by testing for a correlation and reporting the P and r^2. For panels I-L, it looks like there may be some changes but it is difficult to conclusively attribute them to changes in Ih rather than LTP induction more broadly without a pharmacological control. The language discussing these data throughout the paper should reflect this. It may be appropriate to include a summary panel for panel P, like panel O is for panel N.

I believe the experimental unit in the paper is the cell. It may be beneficial to report the numbers of animals in addition to numbers of cells.

It could be useful to report more details on animal husbandry, such as enrichment, temperature and humidity, housing arrangements, lighting schedule.

Supplementary table final row first cell appears to have "b" in italics. It is unclear to me what this refers to.

Discussion mentions that CaMKII is not expressed in interneurons but I don't think this is accurate. For example:

Keaveney et al. 2020, DOI: https://doi.org/10.1523/JNEUROSCI.2570-19.2020

Veres et al. 2023, DOI: 10.1523/ENEURO.0070-23.2023.

Statistics:

The statistical approach is generally reasonable, with 1 and 2 sample t-tests and multi-factor analyses, as well as tests of normality. I felt compelled to select "no" here despite of this, as I believe the statistical reporting is not complete. I would expect the 2-way ANOVA results to contain 2 sets of P values, 1 for each factor. For example, fig 1 panel H, the two factors should be explicitly stated either in text or the legend, drug and current step, and the F statistic, and degrees of freedom should be reported in addition to the P value. The same for other panels with multi-factor tests. These statistics do not appear to be in the supplementary statistics document. In fig 2 g,h and j,k it would be appropriate to present the cumulative frequencies of the mIPSCs, not only the means extracted from individual cells. The more appropriate test for mIPSCs would be the Kolmogorov-Smirnov test to compare the distributions. In fig 3, there appear to be n=2 cells in the strong potentiation group in panel D, which are then used in panels G and H. The measured changes are relatively small at ~1mV and ~5% for sag and ss respectively in panels G and H. It is difficult to comment on the distribution of the data at this power. It is therefore difficult to be confident in the results of the ttests. It would be more convincing with a pharmacological control but I don't think this was done here. Fan et al. 2005, cited in the discussion, I think did have a post-LTP zd7288 group.
